# Supplementary material for: Comprehensive Annotation and Functional Exploration of MicroRNAs in Lettuce
Source: Front Plant Sci. 2021 Dec 24;12:781836. doi: 10.3389/fpls.2021.781836 (PMC8739914; doi:10.3389/fpls.2021.781836)
Supplement: Supplementary file 2 [file Data_Sheet_8.ZIP › GSM2230750.plot/Lsa-miRN1655_Lsat_1_v5_gn_8_102901.1_1500_TPlot.pdf]

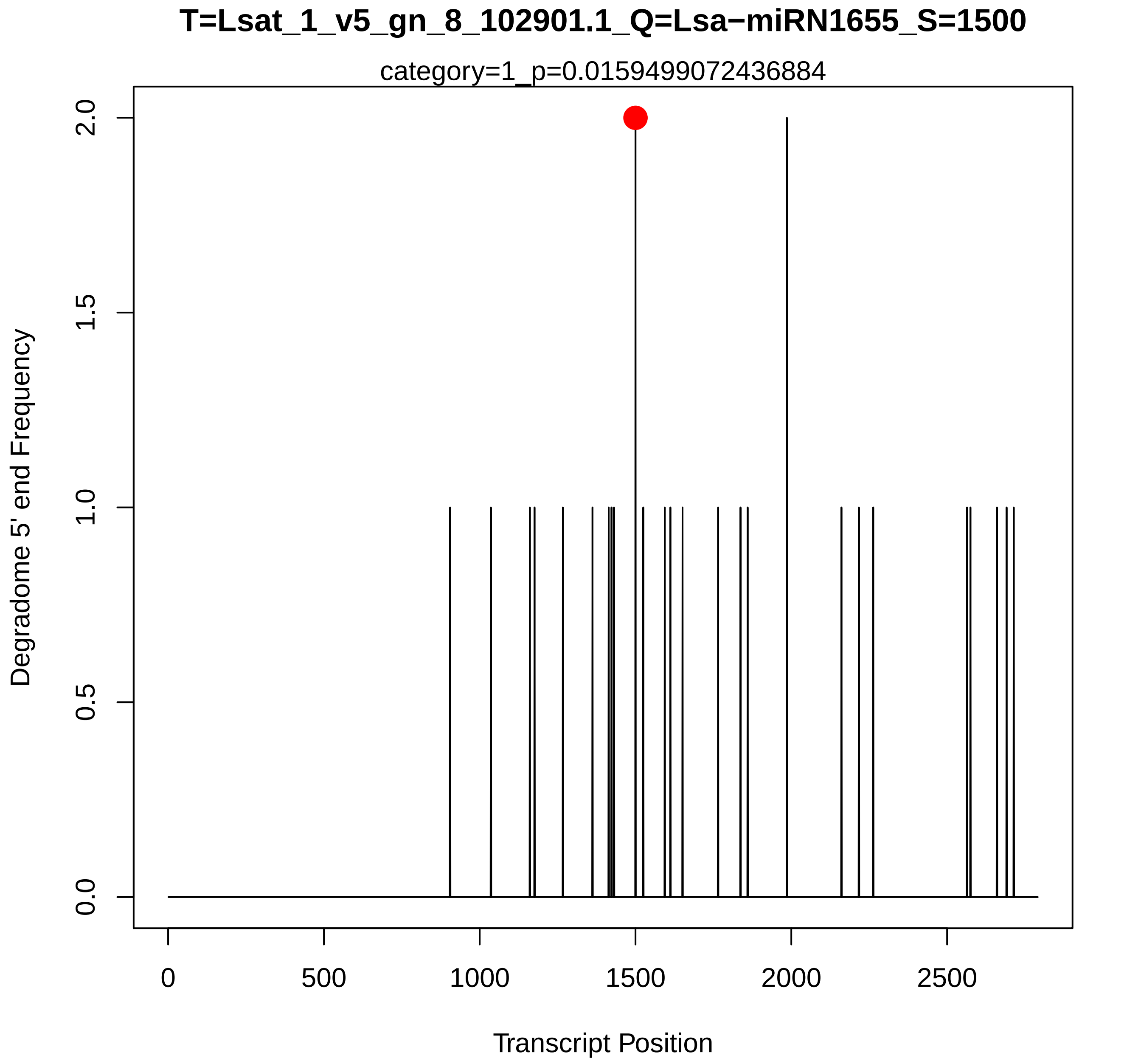
T=Lsat_1_v5_gn_8_102901.1_Q=Lsa−miRN1655_S=1500
category=1_p=0.0159499072436884
Degradome 5' end Frequency
0.0
0.5
1.0
1.5
2.0
0
500
1000
1500
2000
2500
Transcript Position
